# Glyoxalase 1 gene expression in various types of cancer cells immunopathology: a pan-cancer analysis study

**DOI:** 10.3389/fonc.2025.1610886

**Published:** 2025-07-14

**Authors:** Ali M. Alaseem, Jehad A. Aldali, Glowi Alasiri, Muhanad Alhujaily, Khalid I. AlHussaini, Osama A. AlKhamees

**Affiliations:** ^1^ Department of Pharmacology, College of Medicine, Imam Mohammad Ibn Saud Islamic University (IMSIU), Riyadh, Saudi Arabia; ^2^ Department of Pathology, College of Medicine, Imam Mohammad Ibn Saud Islamic University (IMSIU), Riyadh, Saudi Arabia; ^3^ Department of Biochemistry, College of Medicine, Imam Mohammad Ibn Saud Islamic University (IMSIU), Riyadh, Saudi Arabia; ^4^ Department of Internal Medicine, College of Medicine, Imam Mohammad Ibn Saud Islamic University (IMSIU), Riyadh, Saudi Arabia

**Keywords:** tumor microenvironment, metabolic reprogramming, glyoxalase system, GLO-1 expression, cancer progression

## Abstract

Metabolic reprogramming within the tumor microenvironment significantly affects cancer progression by shifting toward aerobic glycolysis and lactate production, while also supporting mitochondrial oxidative phosphorylation. The glyoxalase system, comprising GLO-1 and GLO-2, maintains metabolic homeostasis by neutralizing methylglyoxal (MG) byproducts. GLO-1 protects cells from damage by detoxifying MG via glutathione. In the curent study, pan-cancer analysis revealed elevated GLO-1 mRNA levels across various malignancies, exhibiting variable prognostic implications on patient survival: reduced survival in ACC, MESO, and SARC, and enhanced survival in KIRC and LIHC. GLO-1 activity is regulated by transcriptional and post-translational modifications, including phosphorylation, NO-mediated modification, and glutathionylation. The role of GLO-1 in survival and disease course differs depending on the specific cancer. GLO-1 levels were associated with immunotherapy markers like microsatellite instability (MSI) and tumor mutational burden (TMB), with positive correlations between GLO-1 and MSI in UCEC, TGCT, and STAD, and between GLO-1 and TMB in LUAD, UCEC, LIHC, MESO, SKCM, and READ. In terms of immune cell presence, GLO-1 was associated with increased endothelial and neutrophil cells, decreased T and B cell populations, and increased activated CD4 T cells, memory B cells, and type 2 helper T cells. In summary, our study highlights GLO-1 as a significant biomarker across multiple cancers that plays a key role in cancer progression, immune modulation, and therapeutic response.

## Graphical Abstract

Created in BioRender. ALASEEM, A. (2025) https://BioRender.com/eaefrf3.

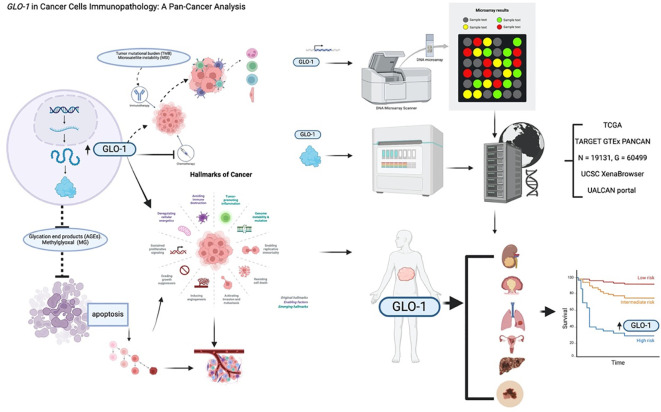

## 1 Introduction

Glyoxalase 1 (GLO-1) is a key enzyme in the glyoxalase system, catalyzing the conversion of methylglyoxal (MG) into S-D-lactoylglutathione, thereby mitigating the formation of advanced glycation end products (AGEs). The GLO-1 gene is located on human chromosome 6p21.2 (1, 2) Reduction in GLO-1 expression leads to the accumulation of MG, triggering apoptosis. To better understand GLO-1’s significance in cancer development, its functional role in cancer progression has been investigated (3). GLO-1 dysregulation has been implicated in various cancers, prompting the explorationof pharmacological and genetic strategies targeting cancer cells via GLO-1 modulation (4–6). GLO-1 is frequently upregulated in multiple malignancies (7, 8). This increased GLO-1 expression is associated with both cancer development and resistance to chemotherapy (9, 10).

The Warburg effect, a hallmark of cancer metabolism characterized by increased glycolysis (11), provides cancer cells with advantages such as enhanced proliferation, migration, survival, and resistance to drug therapies (12). In normal cells, glyoxalase 2 (GLO-2) plays a role in glutathione recycling, contributing to cellular redox balance and protection against oxidative stress. Cancer cells also utilize GLO-2 for similar functions, maintaining redox equilibrium and mitigating oxidative damage (2, 4). A key distinction is that GLO-1 expression levels vary in cancer; often being upregulated, while GLO-2 expression generally remains consistent in both normal and malignant cells. Both GLO-1 and GLO-2 contribute to cellular redox homeostasis, but in cancer cells, an altered redox state can promote survival and resistance to therapy (4). GLO-1 functions as a cellular defense enzyme by detoxifying MG (2). Impaired MG metabolism results in the accumulation of dicarbonyls, reactive metabolites that can interact with nucleotides and proteins, inhibiting cell proliferation and potentially inducing cell death (9, 10).

Studies employing cell culture models, patient samples, and tissue microarray analysis have previously shown that GLO-1 is overexpressed during melanoma progression (13). Furthermore, CRISPR/Cas9-based GLO-1 deletion and rescue experiments have uncovered a novel function for GLO-1 as a molecular regulator of invasion and metastasis in melanoma. This observation aligns with existing evidence demonstrating GLO-1’s control over epithelial-mesenchymal transition (EMT) and metastatic behavior in prostate carcinoma cells (14, 15). The growing body of evidence indicates that dysregulation of GLO-1 expression and activity may be a key factor in cancer initiation, progression, and the development of therapeutic resistance. This research aims to add to the current state of knowledge regarding the association between GLO-1 and cancer, as well as its potential clinical implications (2, 16).

Considering the potential tumorigenic role of GLO-1, we analyzed its pathological and prognostic value in a range of malignancies. Specifically, we investigated whether GLO-1 could serve as a positive prognostic marker and inform the development of more effective therapies, ultimately leading to improved patient outcomes. As a result, the present study provides a systematic evaluation of the GLO-1 gene’s role in cancer progression and development, encompassing an analysis of the impact of genetic modifications and mutations on GLO-1, as well as the protein’s immunotherapeutic function. Our findings establish GLO-1 as a promising prognostic biomarker with potential clinical significance in multiple cancer types.

## 2 Methods

### 2.1 Dataset information

A standardized pan-cancer dataset (TCGA TARGET GTEx, PANCAN, N = 19131, G = 60499), was downloaded from the UCSC XenaBrowser database (https://xenabrowser.net/). Expression data for the GLO-1 gene (ENSG00000124767) was extracted for each sample. Additional analyses were conducted on samples from various tissue types obtained from the database, including solid tissue (normal), primary solid tumor, additional primary tumor samples., normal tissue, primary blood-derived cancer (bone marrow), and primary blood-derived cancer (peripheral blood) (**Supplementary Table S1**).

### 2.2 Gene expression and survival analysis

We compared GLO-1 expression levels across 33 different cancer types (16, 17) using the TIMER2.0 web server and the GEPIA2.0 database. Specifically, the survival Mmp module within GEPIA2.0 was employed to perform survival analysis, assessing the correlation between GLO-1 expression and both overall survival (OS) and disease-free survival (DFS). Samples were stratified into high and low GLO-1 expression groups based on the median expression value.

### 2.3 Pathological stage analysis

We analyzed total GLO-1 protein expression in primary tumors and normal tissues using the Clinical Proteomic Tumor Analysis Consortium dataset accessed through the UALCAN portal (http://ualcan.path.uab.edu/analysis-prot.html) (4). We focused on breast, ovarian, and colon cancer datasets to examine the correlation between GLO-1 protein levels and clinical stages.

### 2.4 Analysis of genetic alterations

We employed the Cancer Genomics Dataset from the TCGA Pan-Atlas project, accessed through the cBioPortal (http://cbioportal.org), to investigate GLO-1 genetic alterations. This resource was utilized to identify mutations within the GLO-1 gene. Furthermore, we analyzed the mutation frequency across 17 cancer types using the TIMER2.0 database. Finally, copy number alteration data for GLO-1 were collected using the SangerBox tool.

### 2.5 Analysis of immune-related gene expression and immune infiltration

To explore the potential interplay between GLO-1 expression and immune regulation within the tumor microenvironment, we analyzed the correlation between GLO-1 expression and a comprehensive panel of 47 immune checkpoint (ICP) genes. This analysis was conducted using SangerBox, an established online tool for accessing and analyzing data from TCGA. Spearman’s rank correlation test was employed to assess the statistical significance of these correlations. Tumor mutational burden (TMB), microsatellite instability (MSI), and the level of immune cell infiltration were considered key determinants of the tumor microenvironment and potential modulators of the observed relationships.

### 2.6 Analysis of drug sensitivity using the gene set cancer analysis database

We investigated the relationship between gene expression and drug sensitivity using the Genomics of Drug Sensitivity in Cancer and Cancer Therapeutics Response Portal (CTRP) datasets, which contain data from cancer cell lines. Pearson correlation analysis was performed to assess the correlation between GLO-1 expression levels and susceptibility to small molecule drugs, as measured by IC50 values. Furthermore, the Tumor Immune Dysfunction and Exclusion algorithm was employed to evaluate the association between GLO-1 expression and response to immunotherapeutic interventions.

## 3 Results

### 3.1 GLO-1 expression and correlation with clinicopathological features in pan-cancer

**Figure 1:**
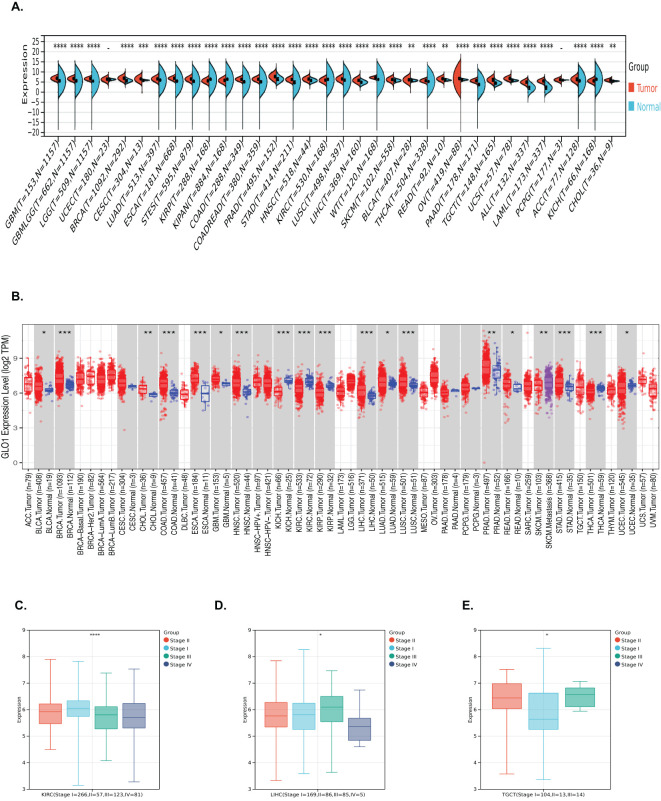
Analysis of GLO-1 mRNA expression in diverse cancer types and stages. **(A, B)** GLO-1 mRNA expression levels were compared between various malignancies and corresponding normal control tissues using SangerBox and TIMER 2.0. **(C–E)** GLO-1 mRNA expression levels are shown for multiple cancer types. Statistical significance is indicated as follows: *p < 0.05; **p < 0.01; ***p < 0.001. **** denote statistical significance of the differences in gene expression between tumor and normal tissues (or among clinical subgroups).

Pan-cancer analysis of GLO-1 expression was performed using the SangerBox and TIMER2.0 databases. Compared to normal tissue, GLO-1 was significantly upregulated in 28 tumors, including GBM (Tumor: 6.73 ± 0.48, Normal: 5.37 ± 1.31, p = 6.7e−75), GBMLGG (Tumor: 6.58 ± 0.41, Normal: 5.37 ± 1.31, p = 8.5e−216), LGG (Tumor: 6.53 ± 0.37, Normal: 5.37 ± 1.31, p = 3.7e−178), BRCA (Tumor: 6.83 ± 0.68, Normal: 5.37 ± 1.31, p = 3.7e−179), EGFR (Tumor: 6.83 ± 0.68, Normal: 5.37 ± 1.31, p = 3.7e−179), CESC (Tumor: 6.44 ± 0.63, Normal: 5.91 ± 0.21, p = 1.2e−4), LUAD (Tumor: 6.49 ± 0.68, Normal: 5.87 ± 0.93, p = 3.5e−42), ESCA (Tumor: 6.78 ± 0.62, Normal: 5.25 ± 1.26, p = 6.3e−84), STES (Tumor: 6.44 ± 0.68, Normal: 5.13 ± 1.38, p = 3.6e−165), COAD (Tumor: 6.40 ± 0.52, Normal: 4.99 ± 1.67, p = 2.6e−90), COADREAD (Tumor: 6.43 ± 0.55, Normal: 5.01 ± 1.66, p = 1.9e−102), PRAD (Tumor: 7.81 ± 0.90, Normal: 6.52 ± 1.03, p = 4.7e−34), STAD (Tumor: 6.29 ± 0.65, Normal: 4.75 ± 1.64, p = 6.8e−64), HNSC (Tumor: 6.49 ± 0.60, Normal: 5.72 ± 0.54, p = 9.7e−15), LUSC (Tumor: 6.52 ± 0.66, Normal: 5.87 ± 0.93, p = 9.2e−48), LIHC (Tumor: 5.86 ± 0.76, Normal: 4.87 ± 0.60, p = 9.8e−38), WT (Tumor: 7.21 ± 0.38, Normal: 6.07 ± 1.49, p = 3.5e−35), SKCM (Tumor: 6.14 ± 0.83, Normal: 5.78 ± 0.39, p = 4.1e−11), BLCA (Tumor: 6.13 ± 0.74, Normal: 5.78 ± 0.33, p = 1.3e−3), THCA (Tumor: 5.64 ± 0.43, Normal: 5.29 ± 0.92, p = 7.8e−28), READ (Tumor: 6.50 ± 0.62, Normal: 6.02 ± 0.34, p = 4.8e−3), OV (Tumor: 6.59 ± 1.08, Normal: 6.11 ± 0.26, p = 5.6e−14), PAAD (Tumor: 5.58 ± 0.46, Normal: 3.62 ± 1.58, p = 1.0e−53), TGCT (Tumor: 6.01 ± 0.89, Normal: 4.47 ± 0.63, p = 1.3e−37), and UCS (Tumor: 6.72 ± 0.60, Normal: 5.74 ± 0.29, p = 5.2e-18). GLO-1 was significantly downregulated in KIRP (Tumor: 5.58 ± 0.68, Normal: 6.07 ± 1.49, p = 3.2e−21), KIPAN (Tumor: 5.74 ± 0.67, Normal: 6.07 ± 1.49, p = 3.0e−18), KIRC (Tumor: 5.86 ± 0.63, Normal: 6.07 ± 1.49, p = 5.5e−12), and KICH (Tumor: 5.54 ± 0.70, Normal: 6.07 ± 1.49, p = 4.2e−10) (**Figures 1A, B**). The association between GLO-1 expression and cancer stage was also assessed, revealing significant stage-dependent differences in BLCA (Stage I = 266, II = 57, III = 123, IV = 81, p = 2.4e−5), LIHC (Stage I = 169, II = 86, III = 85, IV = 5, p = 0.03), and TGCT (Stage I = 104, II = 13, III = 14, p = 0.02) (**Figures 1C–E**).

### 3.2 Impact of GLO-1 on cancer progression in pan-cancer

**Figure 2:**
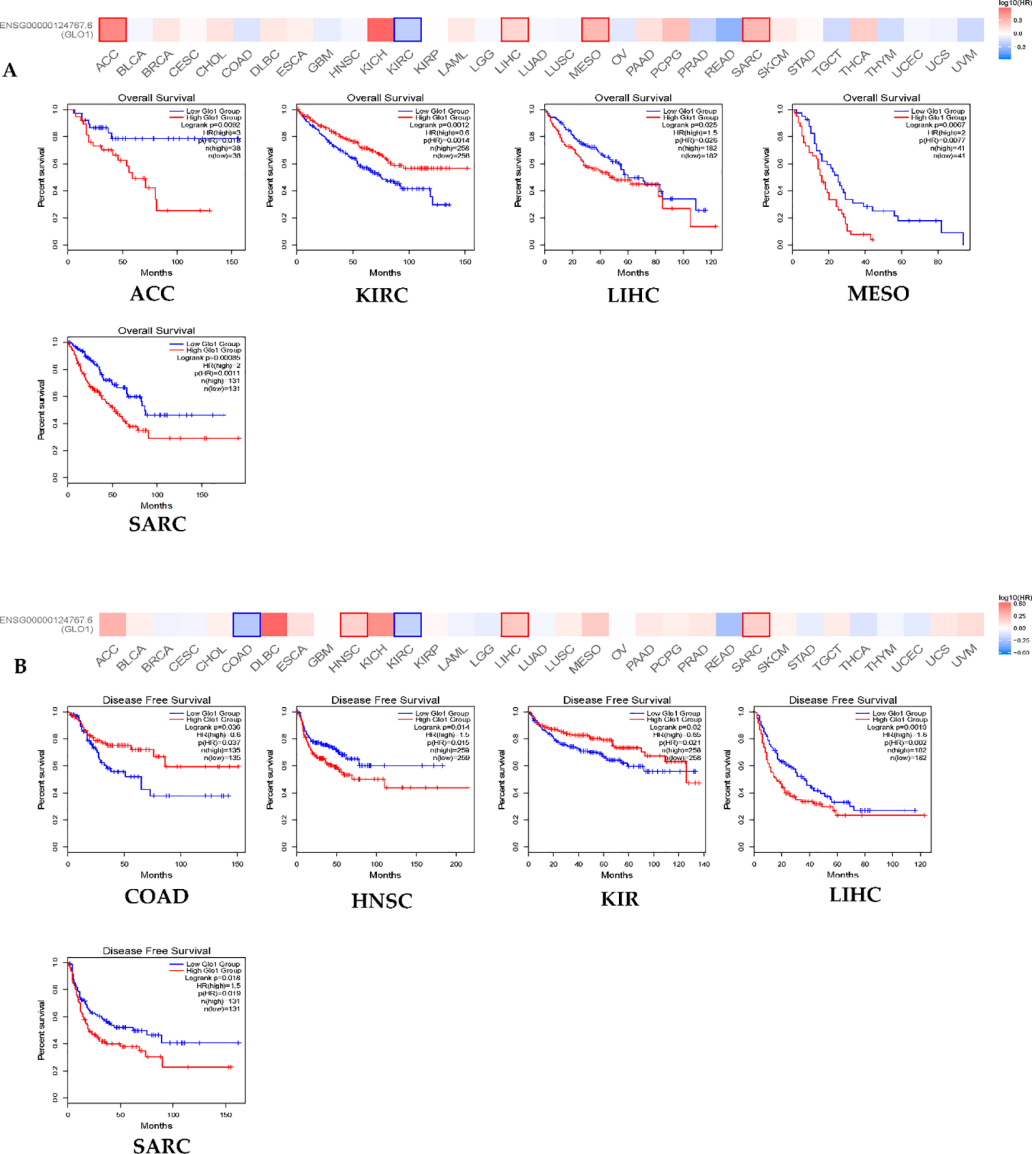
GLO-1 expression and TCGA cancers. **(A)** overall survival (OS) and **(B)** disease-free survival (DFS) were conducted using the GEPIA2 online tool, stratifying TCGA tumor samples based on GLO1 gene expression levels. The survival map and positive Kaplan-Meier curves are presented.

To investigate the prognostic value of GLO-1 across multiple cancers, we analyzed its impact on both overall survival (OS) and disease-free survival (DFS) in the pan-cancer cohort. Our findings revealed that elevated GLO-1 expression correlated with poorer OS in ACC, MESO, and SARC, but no such association was observed in KIRC or LIHC (**Figure 2A**). Furthermore, we confirmed that higher GLO-1 expression was significantly associated with reduced DFS in HNSC, SARC, and LIHC, but not in KIRC or COAD (**Figure 2B**).

### 3.3 Analysis of genetic and epigenetic alterations

**Figure 3:**
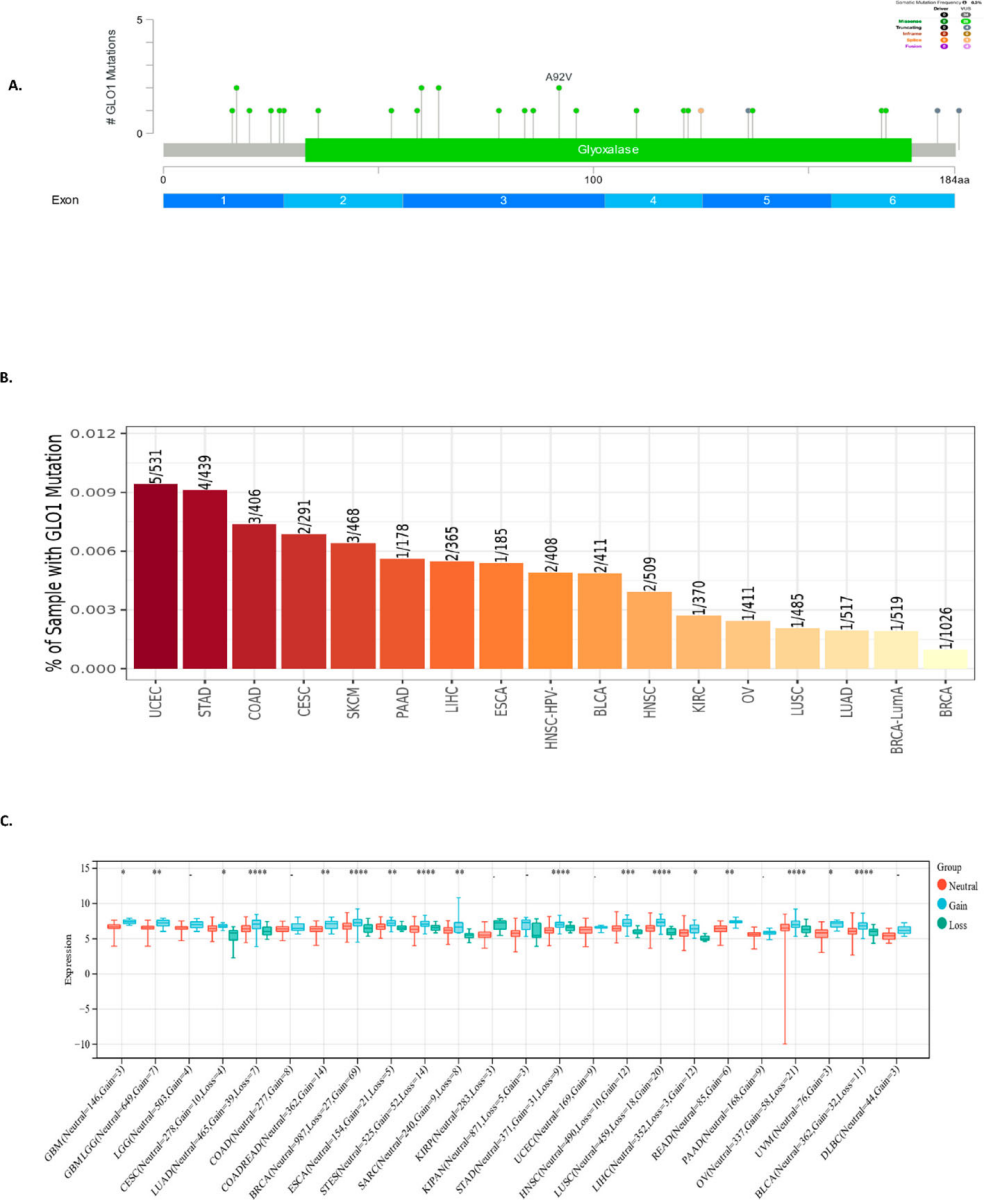
Analysis of GLO-1 genetic alterations in cancer. **(A)** Schematic representation of GLO-1 mutations, with indicated mutation types. **(B)** GLO-1 mutation rates across various TCGA cancer tissues. **(C)** GLO-1 copy number variation (CNV) analysis in various malignancies. Statistical significance is indicated as follows: *p < 0.05; **p < 0.01; ***p < 0.001; ****p < 0.0001.

To characterize the spectrum of GLO-1 genetic alterations, we employed multiple bioinformatic resources. The cBioPortal database was queried to identify the types of GLO-1 mutations, revealing a predominance of missense mutations (**Figure 3A**). Mutation frequencies across various tumor tissues were then determined using the TIMER2.0 database, highlighting the highest rates in UCEC (5/531), STAD (4/439), and COAD (3/406) (**Figure 3B**). To assess the impact of copy number variations (CNVs) on GLO-1 genomic status, we utilized the SangerBox database. Statistical analysis revealed significant differences in CNV profiles across 17 distinct tumor types, including glioblastoma multiforme (Neutral = 146, Gain = 3, p = 0.03), GMBLGG (Neutral = 649, Gain = 7, p = 6.2e−3), CESC (Neutral = 278, Gain = 10, Loss = 4, p = 0.02), LUAD (Neutral = 465, Gain = 39, Loss = 7, p = 8.3e−5), COADREAD (Neutral = 362, Gain = 14, p = 3.9e−3), BRCA (Neutral = 987, Loss = 27, Gain = 69, p = 7.3e−8), ESCA (Neutral = 154, Gain = 21, Loss = 5, p = 2.6 e−3), STES (Neutral = 525, Gain = 52, Loss = 14, p = 5.3e-11), SARC (Neutral = 240, Gain = 9, Loss = 8, p = 4.2e−3), STAD (Neutral = 371, Gain = 31, Loss = 9, p = 3.9e−8), HNSC (Neutral = 490, Loss = 10, Gain = 12, p = 1.6e−4), LUSC (Neutral = 459, Loss = 18, Gain = 20, p = 1.4e−7), LIHC (Neutral = 352, Loss = 3, Gain = 12, p = 0.02), READ (Neutral = 85, Gain = 6, p = 1.1e−3), OV (Neutral = 337, Gain = 58, Loss = 21, p = 3.7e−6), UVM (Neutral = 76, Gain = 3, p = 0.03), and BLCA (Neutral = 362, Gain = 32, Loss = 11, p = 7.6e−6) (**Figure 3C**).

**Figure 4:**
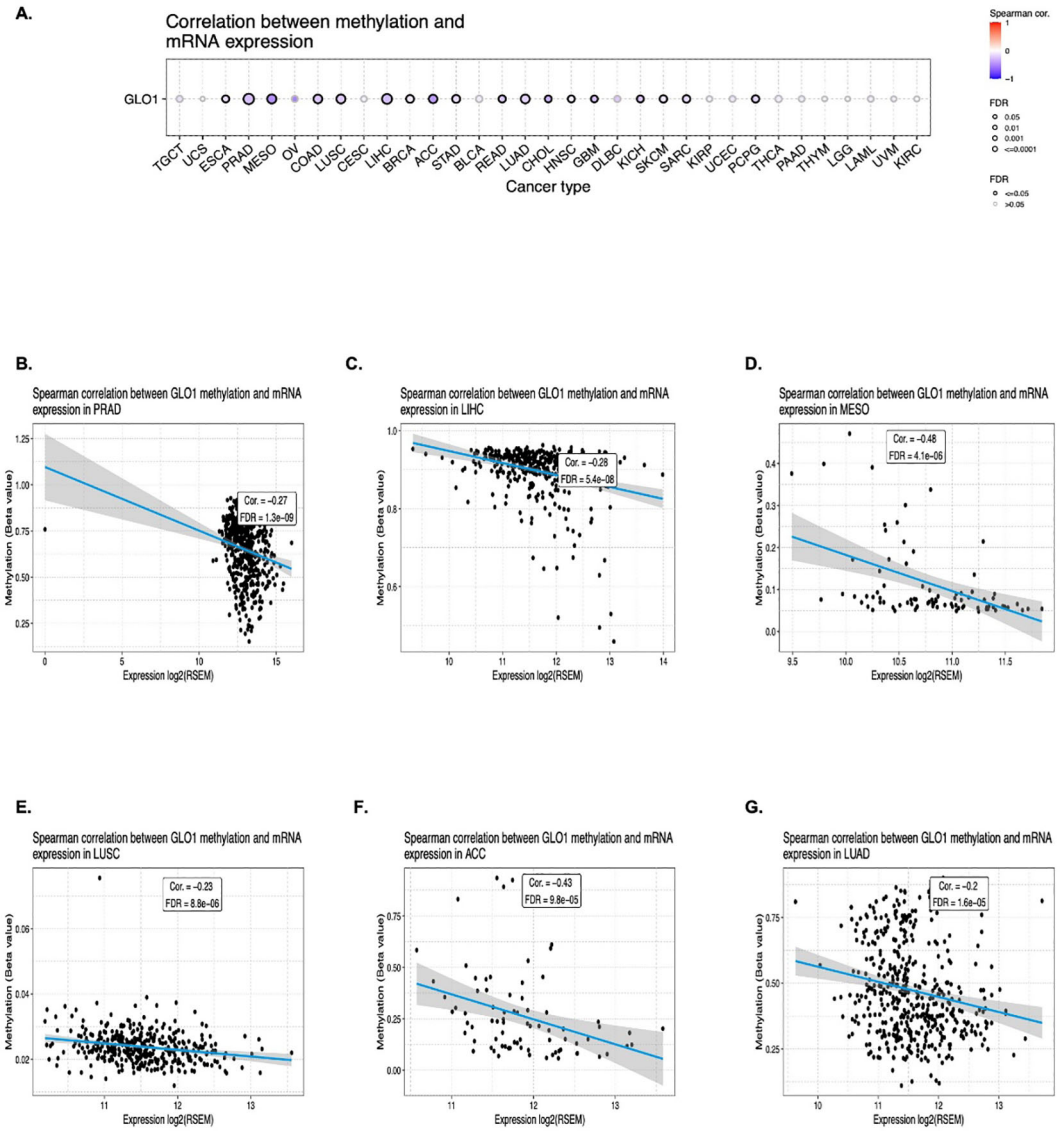
Correlation between GLO-1 mRNA expression and DNA methylation. **(A)** Differential methylation of GLO-1 in tumor versus normal samples across various cancers. Red dots indicate increased methylation in tumors, and blue dots indicate decreased methylation. Correlation between GLO-1 mRNA expression and methylation in **(B)** PRAD, **(C)** LIHC, **(D)** MESO, **(E)** LUSC, **(F)** ACC, and **(G)** LUAD.

To further investigate the potential regulatory mechanisms influencing GLO-1 expression in cancer, we examined DNA methylation alterations as a key epigenetic modification. Using the Gene Set Cancer Analysis (GSCA) database, a comprehensive resource for exploring gene-set enrichment and pathway analysis in cancer, we identified a negative correlation between GLO-1 expression and 18 distinct cancer types. These included ESCA, PRAD, MESO, COAD, LUSC, LIHC, BRCA, STAD, ACC, READ, LUAD, CHOL, HNSC, GBM, KICH, SKCM, SARC, and PCPG (**Figures 4A, B**).

### 3.4 Correlation of GLO-1 gene expression with immunotherapy response

**Figure 5:**
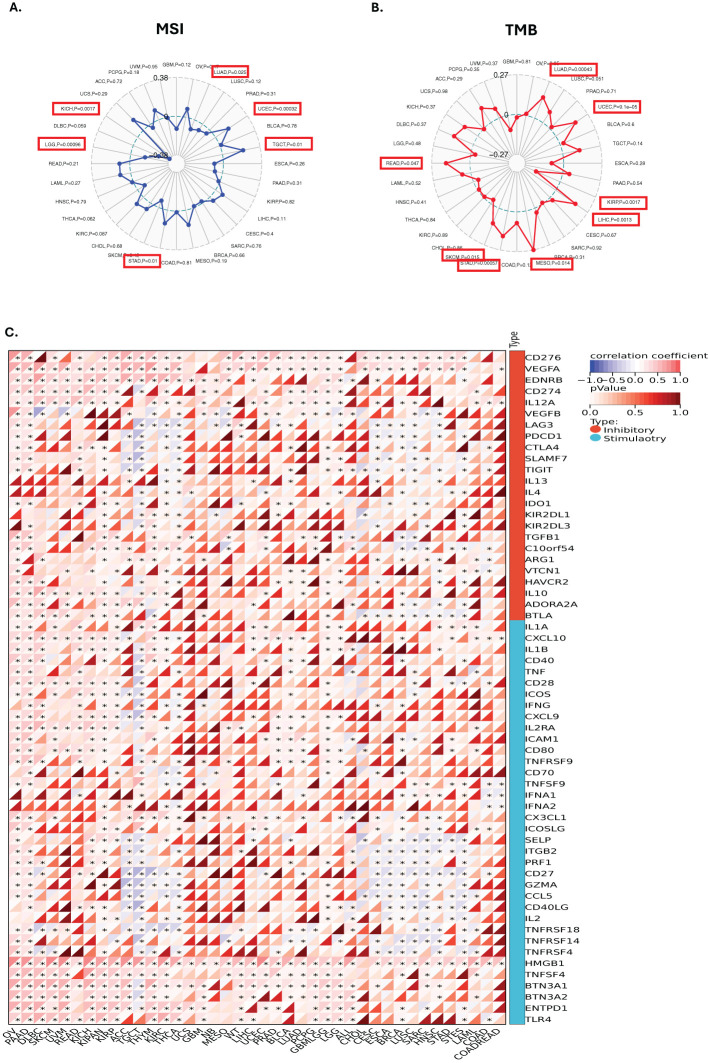
Correlations between GLO-1 expression and immune markers, tumor mutational burden (TMB), and microsatellite instability (MSI). Correlation between GLO-1 expression and **(A)** MSI, **(B)** TMB, and **(C)** immune checkpoint genes. In **(A)** and **(B)**, the range of values is indicated in black, and the correlation coefficient (r) is shown by the blue and red lines. Statistical significance is indicated as follows: *p < 0.05.

To elucidate the potential role of GLO-1 in modulating the response to cancer immunotherapy, we analyzed the correlation between GLO-1 expression and key immunotherapeutic biomarkers: TMB, MSI, and a panel of ICP. We observed a positive correlation between GLO-1 expression and MSI in UCEC, TGCT, and STAD, while a negative correlation was found in LUAD, LGG, and KICH (**Figure 5A**). GLO-1 expression also showed a positive correlation with TMB in several tumor types, including LUAD, UCEC, LIHC, MESO, STAD, SKCM, and READ (**Figure 5A**). However, a negative correlation between GLO-1 and TMB was observed specifically in KIRP tissue (**Figure 5B**). Additionally, GLO-1 expression demonstrated strong associations with both inhibitory and stimulatory ICPs, such as CD276, VEGFA, and HMGB1, across a majority of the analyzed cancer tissues. (**Figure 5C**).

### 3.5 GLO-1 expression and immune cell infiltration

**Figure 6:**
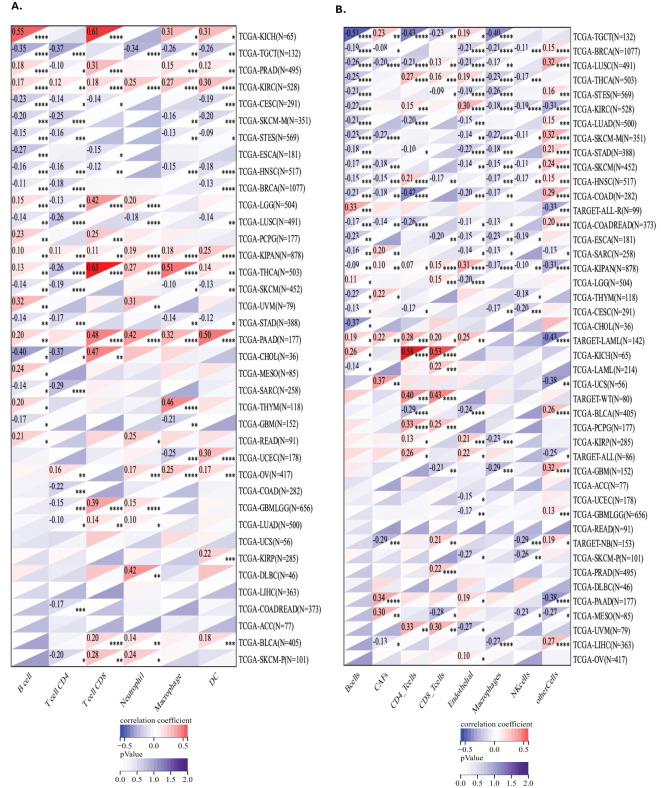
Analysis of GLO-1 expression and immune cell infiltration in cancer using TIMER and EPIC databases. **(A)** Correlation between GLO-1 expression and the abundance of various immune cell types across 38 cancer types, as determined by the TIMER database. **(B)** Correlation between GLO-1 expression and immune cell infiltration across 41 of 43 cancer types, as determined by the EPIC algorithm.

To investigate the relationship between GLO-1 expression and immune cell infiltration, we employed two distinct computational approaches: the TIMER database and the EPIC algorithm (**Figure 6**). Using TIMER, we identified significant correlations between GLO-1 expression and immune infiltration in 19 of the 35 analyzed tumor types (**Figure 6A**). These significant correlations were observed in various cancer types, including TCGA-LUSC, TCGA-MESO, TCGA-OV, TCGA-PAAD, TCGA-PCPG, TCGA-PRAD, TCGA-READ, TCGA-SARC, TCGA-SKCM-M, TCGA-SKCM-P, TCGA-SKCM, TCGA-STAD, TCGA-STES, TCGA-TGCT, TCGA-THCA, TCGA-THYM, TCGA-UCEC, and TCGA-UVM. Analysis using the EPIC algorithm revealed substantial correlations between GLO-1 expression and immune infiltration in a larger set of 41 cancer types (**Figure 6B**). These included TCGA-UVM, TCGA-PAAD, TCGA-TGCT, TCGA-UCS, TCGA-LAML, TARGET-ALL, TCGA-PCPG, TARGET-ALL-R, TCGA-KICH, and TCGA-CHOL.

### 3.6 Drug sensitivity analysis

**Figure 7:**
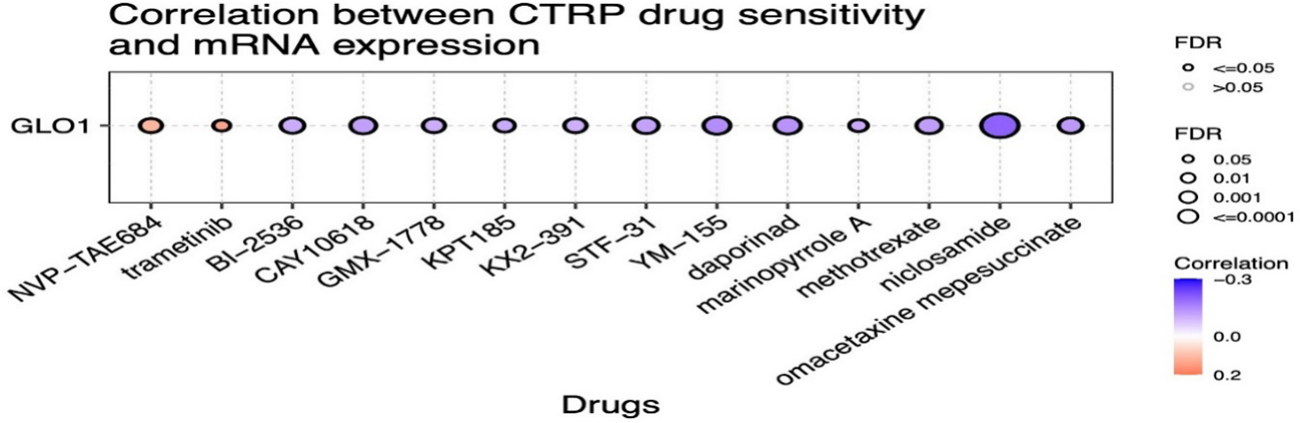
Analysis of the correlation between GLO-1 expression and drug sensitivity using the Cancer Therapeutics Response Portal (CTRP) database. Red dots indicate increased drug sensitivity and blue dots indicate decreased drug sensitivity associated with higher GLO-1 expression.

We utilized the CTRP database to investigate the relationship between GLO-1 expression and sensitivity to a range of therapeutic agents. Our analysis revealed negative correlations between GLO-1 expression and several treatments, including omacetaxine, niclosamide, methotrexate, marinopyrole A, aporina, YM-155, STF-31, KX2-391, KPT185, GMX-1778, CAY-10618, and BI-2536 (**Figure 7**). Conversely, positive correlations were observed between GLO-1 expression and trametinib and NVP-TAE684 (**Figure 7**). Additionally, we explored the potential of GLO-1 expression to predict response to PD1 immunotherapy, finding significant associations in melanoma, glioblastoma, kidney cancer, and bladder cancer (**Table 1**).

**Table 1:** Correlation between GLO-1 expression and response to immunotherapy in clinical studies.

Cohort	Cancer	Survival	Risk	Count
Liu 2019_PD1	Melanoma	OS	3.552	74
Riaz 2017_PD1	Melanoma	OS	2.011	26
Zhao 2019_PD1	Glioblastoma	OS	1.413	9
Zhao 2019_PD1	Glioblastoma	PFS	0.899	9
Liu 2019_PD1	Melanoma	PFS	1.384	74
Gide2019_PD1 + CTLA4	Melanoma	OS	1.325	32
Zhao2019_PD1	Glioblastoma	PFS	0.821	15
Miao2018_ICB	Kidney	PFS	1.074	33
Gide2019_PD1 + CTLA4	Melanoma	PFS	0.994	32
Riaz2017_PD1	Melanoma	PFS	1.318	26
Gide2019_PD1	Melanoma	OS	0.61	41
VanAllen2015_CTLA4	Melanoma	OS	1.267	42
Zhao2019_PD1	Glioblastoma	OS	0.304	15
VanAllen2015_CTLA4	Melanoma	PFS	0.711	42
Gide2019_PD1	Melanoma	PFS	0.666	41
Miao2018_ICB	Kidney	OS	−0.08	33
Liu2019_PD1	Melanoma	PFS	0.207	47
Lauss2017_ACT	Melanoma	OS	−0.027	25
Liu2019_PD1	Melanoma	OS	−0.031	47
Nathanson2017_CTLA4	Melanoma	OS	0.891	15
Braun2020_PD1	Kidney	PFS	−0.468	295
Braun2020_PD1	Kidney	OS	−0.443	295
Hugo2016_PD1	Melanoma	OS	−0.758	25
Lauss2017_ACT	Melanoma	PFS	−0.429	25
Mariathasan2018_PDL1	Bladder	OS	−1.936	348
Riaz2017_PD1	Melanoma	OS	−1.686	25
Nathanson2017_CTLA4	Melanoma	OS	−2.048	9
Riaz2017_PD1	Melanoma	PFS	−2.116	25

## 4 Discussion

**Figure 8:**
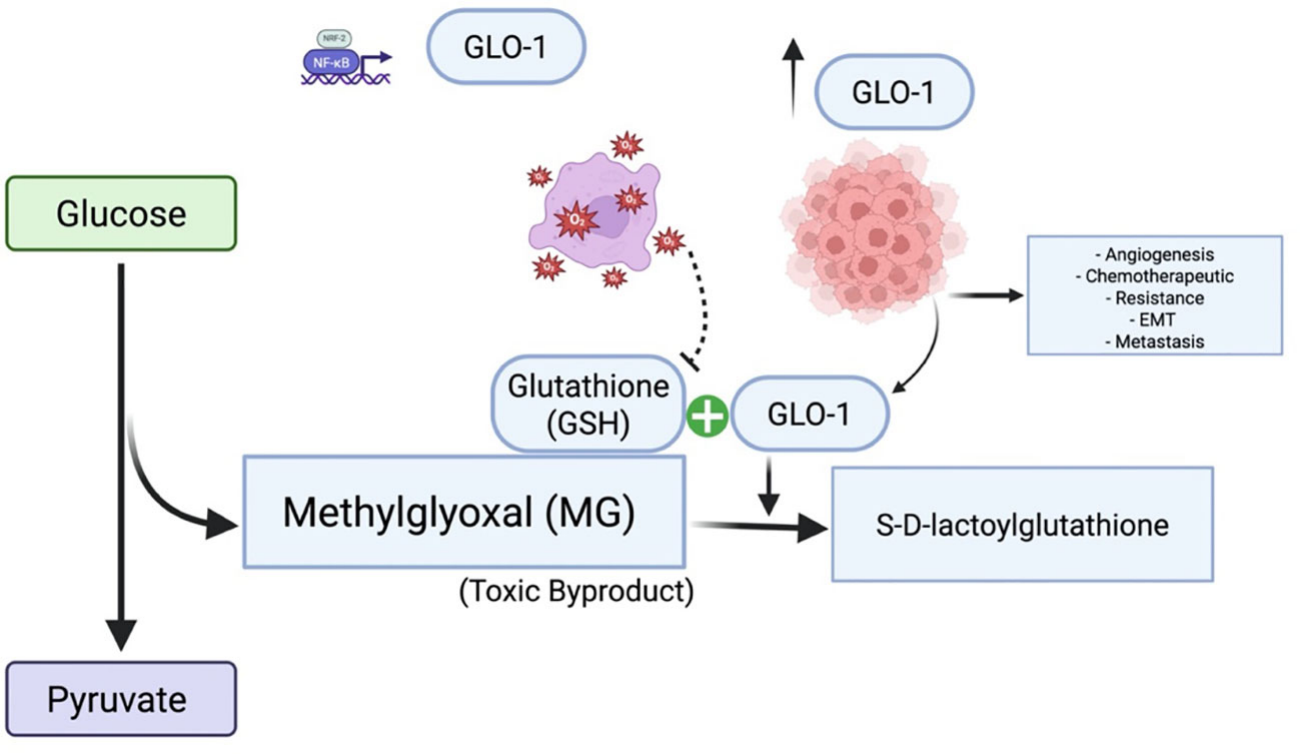
Glyoxalase 1 (GLO1) in cancer: mechanisms and therapeutic targeting. GLO1 promotes tumor cell survival by detoxifying MG, a cytotoxic byproduct of enhanced glycolytic metabolism. Overexpression of GLO1 facilitates chemoresistance by preventing MG-induced DNA damage and apoptosis, highlighting its potential as a therapeutic target. Under conditions of GSH depletion and oxidative stress, GLO1 overexpression paradoxically contributes to MG accumulation, thereby promoting cancer development and progression. Created in BioRender. Alaseem, A. (2025) https://BioRender.com/9vf13o0.

Metabolism dysregulation, a characteristic feature of numerous metabolic disorders, is also recognized as a significant driver of tumorigenesis (17). The uncontrolled proliferation inherent in neoplastic transformation necessitates not only the disruption of cell cycle control but also a coordinated reprogramming of cellular metabolism to sustain this aberrant growth—a concept recognized as a hallmark of cancer over a decade ago (18). Aerobic glycolysis, a fundamental metabolic adaptation in cancer cells, promotes the preferential fermentation of glucose to lactate through a process that also generates MG (18). While the association between aerobic glycolysis and cancer cell proliferation is well established, recent findings have demonstrated that metabolism plays a broader, crucial role in shaping diverse cellular functions through highly dynamic and adaptable processes (19). Such metabolic reprogramming is essential for tumor development, progression, and immune evasion, ultimately contributing to the establishment of an immunosuppressive microenvironment (**Figure 8**). Therefore, understanding the genes involved in this metabolic rewiring within tumors and their surrounding microenvironment is of critical importance (20). MSI and TMB have been identified as established immune predictive scores capable of estimating immunotherapy therapeutic efficacy and predicting treatment response (21, 22). Our pan-cancer analysis of GLO-1 revealed differential expression across various cancer stages, suggesting its potential as both a tumorigenic and prognostic marker. This analysis further revealed a complex interplay between GLO-1 and the tumor immune landscape. Specifically, we observed positive correlations between GLO-1 and both TMB and MSI in UCEC, TGCT, and STAD, but negative correlations in LUAD, LGG, and KICH. We also found a positive correlation between GLO-1 expression and TMB in LUAD, UCEC, LIHC, MESO, STAD, SKCM, and READ, with a contrasting negative correlation in KIRP tissue.

In tumor cells exhibiting high rates of aerobic glycolysis, increased expression of GLO-1 appears to be a crucial adaptation, enhancing the detoxification of MG and contributing to resistance against multiple therapeutic agents (**Figure 8**) (23). Many antitumor drugs exploit the elevated MG levels characteristics of cancer cells to induce cytotoxicity and inhibit proliferation. The hypoxic conditions often present within tumor microenvironments further potentiate GLO-1 activity, thereby exacerbating this resistance mechanism (24). Thus, comprehensive insights into the intricate interplay between aerobic glycolysis and the glyoxalase system in cancer are essentialfor the development of novel therapeutic interventions (25).GLO-1 is emerging as a compelling therapeutic target, particularly in glycolysis-dependent tumors. Inhibition of GLO-1 impairs MG detoxification, leading to intracellular dicarbonyl stress that compromises cancer cell survival. In addition to small-molecule inhibitors, RNA interference techniques such as siRNA offer effective strategies for reducing GLO-1 expression. Emerging tools like CRISPR-Cas9 may enable precise modulation of GLO-1, providing opportunities for more targeted and controlled therapeutic interventions.

While direct inhibition of tumor glycolysis has been proposed as a seemingly straightforward strategy to deprive cancer cells of energy (25), studies targeting complex regulatory networks of glycolysis-associated genes have revealed a paradoxical increase in glycolytic gene expression, suggesting the existence of negative feedback mechanisms activated by tumor cells that can lead to unanticipated therapeutic outcomes (26, 27). Moreover, the inherent heterogeneity of tumors, even within a single tumor mass, contributes to the presence of diverse metabolic patterns and regulatory mechanisms, further complicating therapeutic strategies (26, 28). The glyoxalase system, of which GLO-1 is a key component that facilitates the glutathione (GSH)-dependent detoxification of MG, thereby mitigating cellular damage induced by endogenous cytotoxic metabolites (29). In hyperglycemic states, such as those observed in diabetes mellitus, MG production is significantly elevated. The efficient detoxification activity of GLO-1 helps maintain relatively lower plasma MG levels compared to glucose, underscoring its importance in preventing MG-mediated cellular damage and subsequent complications (30). Consequently, enhancing GLO-1 activity or expression in diabetic patients has been proposed as a therapeutic strategy to prevent and treat diabetes-associated complications. For instance, it has been shown the therapeutic potential of a biocompatible scaffold incorporating genes for both GLO-1 and β-klotho to reduce MG levels and reprogram diabetic adipose-derived stem cells (dADSCs) for the treatment of diabetic foot ulcers (31). Another study found that GLO-1 overexpression in dADSCs restored proangiogenic capacity in diabetic ischemic models (32). An imbalance in MG/GLO-1 homeostasis contributes to diabetic vascular complications, including MG-induced impaired neovascularization (33). In a related study, targeted overexpression of GLO-1 specifically in bone marrow cells of diabetic mice was shown to effectively restore neovascularization in ischemic tissues by protecting these cells from MG-induced damage (34). While this approach demonstrates therapeutic potential in the context of wound healing and diabetic complications, it raises concerns regarding potential pro-tumorigenic effects and contributions to cancer’s adaptive mechanisms. However, given the apparent protective role of GLO-1 under certain conditions, direct targeting of GLO-1 alone may not represent the optimal therapeutic strategy. This context-dependent role of GLO-1 in cancer necessitates further investigation into the underlying mechanisms and its therapeutic potential in this context. Emerging evidence supports the combination of GLO-1 inhibitors with immune modulators. Our findings suggest that combining immunotherapy with therapies targeting GLO-1 may offer a synergistic therapeutic benefit. Specifically, GLO-1 inhibition could potentially enhance the effectiveness of immunotherapy by creating a more favorable tumor microenvironment conducive to immune cell infiltration and cytotoxic activity. Furthermore, associations between GLO-1 and immunoregulatory markers as PD-L1, TMB, and MSI strongly support exploring combination therapies with immune checkpoint blockade to achieve enhanced synergistic effects. The clinical outcome of novel GLO-1 inhibitors will likely depend on tumor type, expression levels, immune infiltration, and treatment context. Preclinical validation of these combination strategies is essential to translate this bioinformatics analysis into clinical applications. A promising approach may involve the synergistic inhibition of both GLO-1 and MG, which could also positively impact other comorbidities. While such a combined approach might compromise GLO-1’s detoxification function the overall effect could ultimately be detrimental to cancer development.

Our observation of a positive correlation between GLO-1 and endothelial cells aligns with previous findings demonstrating the reliance of endothelial cells on aerobic glycolysis during blood vessel formation, suggesting a potential interplay between endothelial cell metabolism and cancer cell metabolism (35). This metabolic adaptation in endothelial cells may contribute to angiogenesis, a well-established hallmark of cancer associated with numerous negative prognostic factors in cancer pathology (18). This process can be particularly beneficial for solid tumors, facilitating their sustained growth and promoting metastasis to both adjacent and distant organs. This context may also contribute to the variable (pleiotropic) expression of GLO-1 observed across different cancer types and stages. Metabolic reprogramming in the tumor microenvironment plays a crucial role in cancer cell survival and progression. However, a functional screen in liver cancer identified GLO-1 as a potential tumor suppressor gene (36). Our study suggests that the observed negative correlation of GLO-1 in certain cancer types may represent an additional contributing mechanism. In solid tumors, glycolysis may serve functions beyond energy production. Consistent with this notion, studies in mouse models have shown that knockdown of GLO-1 using shRNA constructs leads to enhanced tumor growth, further supporting a complex and context-dependent role for GLO-1 in tumorigenesis and cancer progression (37). Indeed, GLO-1 appears to function as a dual mediator, exhibiting both oncogenic and tumor-suppressive activities. This functional dichotomy may be attributed to the specific context of the cancer type, including the underlying genetic background of the tumor cells and, importantly, their capacity for MG detoxification. Cancer cells with varying MG detoxification rates could therefore exhibit differential responses to MG-induced stress (14).

Multiple regulatory mechanisms, both transcriptional and post-translational, modulate GLO-1 activity. The GLO-1 promoter region contains multiple regulatory elements that allow transcription factors like AP-2α, AP-1, Nrf2, E2F4, and NF-κB to enhance its activity, leading to increased GLO-1 expression. Additionally, GLO-1 undergoes post-translational modifications, including phosphorylation, nitric oxide-mediated changes, and glutathionylation, further influencing its function and response to cellular conditions (14). The observed data may be influenced by these post-translational modifications, necessitating further detailed investigation to fully elucidate their roles. Our findings suggest that GLO1 methylation as a potential biomarker for gene expression regulation in specific cancers. The tumor-specific variability in GLO1 methylation may also explain differences in clinical outcome, such as immune evasion or therapeutic response.

Our previous studies have demonstrated the upregulation of GLO-1 expression in human malignant melanoma tissue. Furthermore, we found that melanoma cells transfected with siGLO-1 exhibited increased vulnerability to the cytotoxic effects of endogenously produced MG. We also observed a bimodal function of GLO-1 in the regulation of cellular carbonyl stress associated with MG addiction. In a separate study employing CRISPR/Cas 9-mediated deletion of GLO-1 in both *in vivo* and *in vitro* models, we established a novel function for GLO-1 in promoting melanoma cell invasiveness and metastasis. Despite these findings, the clinical significance of GLO-1 expression and its role in cancer immunopathology remains relatively unexplored (14, 15). Although some studies have shown that solid tumors with elevated glucose metabolism exhibit high glycolytic rates and consequently increased MG formation, it has also been reported that, in certain contexts, the accumulation of cytotoxic MG can lead to cell growth inhibition through apoptosis in cancer cells. Consistent with this, higher MG accumulation and increased apoptosis induction have been observed upon GLO-1 knockdown (38). In the present study, we evaluated the expression of the immune checkpoints CD276, CD44, TNFRSF14, and VSIR across a range of cancer tissues. Our analysis revealed a positive correlation between GLO-1 and CD276 and CD44, while TNFRSF14 and VSIR showed a negative correlation with GLO-1. Moreover, it has been shown that GLO-1 contributes to the maintenance of an immunosuppressive microenvironment through MG-H1-mediated upregulation of the immune checkpoint programmed death ligand 1 (PD-L1), thereby promoting cancer progression (39).

The results of the present study demonstrate an association between GLO-1 overexpression and advanced cancer stages, including breast cancer. This finding is supported by a recent investigation into the correlation between GLO-1 and PKCλ expression levels in human breast cancer, which also examined their combined influence on the prognosis of patients with late-stage disease. This study concluded that GLO-1 and PKCλ could serve as potentially effective therapeutic targets for the treatment of late-stage human breast cancer (40). In contrast, another report employing immunohistochemistry to evaluate GLO-1 expression in breast cancer found no significant difference between ductal carcinoma *in situ* and invasive tumors, with the majority of tumor samples exhibiting a GLO-1-IRS greater than 7, a value considered an appropriate cutoff for survival analysis (41). In the context of prostate cancer, a positive correlation between GLO-1 expression levels and both pathological grade and proliferation rate has been reported, suggesting that GLO-1 may function as a risk factor for prostate cancer growth and disease progression (42). A potential role for GLO-1 in the progression of colorectal cancer has also been indicated in other studies (43). In agreement with these findings across various cancer types, our study revealed that disease-free survival was significantly reduced in patients with high GLO-1 expression in HNSC, SARC, and LIHC, but not in KIRC or COAD.

GLO-1 has been implicated in protecting cancer cells from the cytotoxic effects of anticancer drugs in various malignancies (44). In the context of metastatic prostate cancer, elevated GLO-1 expression can contribute to the establishment of an immunosuppressive microenvironment, promoting the upregulation of programmed death ligand 1 (PD-L1) expression, a process mediated by 5-hydro-5-methylimidazolone. This, in turn, further exacerbates the progression of cancer tumors. A direct correlation between GLO-1 and PD-L1 expression levels has been confirmed in tissue samples obtained from patients with prostate cancer. Remarkably, the majority of GLO-1-positive samples exhibited high PD-L1 expression, which was associated with enhanced tumor aggression, invasion, and expansion, leading to metastasis and an increased likelihood of recurrence (39). Our findings provide further evidence supporting the potential of GLO-1 as both a valuable prognostic marker and a promising therapeutic target in cancer. While current clinical data supporting its clinical significance are limited, the established underlying molecular mechanisms suggest potential applications across a broad range of cancers, including non-solid tumors. This also suggests a potential role for GLO-1 in influencing therapeutic responses to various treatments and its potential interactions with immunotherapy agents. However, the variations observed in our study indicate that the therapeutic effectiveness of targeting GLO-1 may be dependent on the specific cancer type and its molecular context.

### 4.1 Study strengths and limitations

While this study provides valuable insights into GLO-1’s role in cancer, it has certain limitations that should be acknowledged. The potential pathways implicated in GLO-1’s function, including signaling regulation, maintenance of stemness, RNA alteration, and control of the tumor microenvironment, have been identified primarily through bioinformatic analysis and require further experimental validation using wet-lab approaches. Furthermore, a more comprehensive analysis of the correlation between GLO-1 mRNA and protein levels is necessary to fully elucidate the observed differences in expression. Additionally, it is crucial to unravel the involvement of GLO-1 in mediating response and resistance to chemotherapy, as numerous studies have highlighted the significant impact of drug tolerance development on treatment outcomes. Although our analyses demonstrate notable associations between GLO-1 expression and several cancer-related features, it is important to recognize that these findings remain correlative and do not establish causality. Therefore, validating these findings requires integrated *in vitro*, *in vivo*, and *in silico* studies. High-throughput *in silico* docking and structure-based drug design offer powerful approaches to accelerate the discovery of clinically relevant GLO-1 inhibitors. Future work should focus on identifying and optimizing small-molecule GLO-1 inhibitors that exploit tumor metabolic dependency on glyoxalase detoxification. These inhibitors should be rationally designed to synergize with other therapeutic modalities, improving selectivity and therapeutic profiles. Such efforts are crucial to complement preclinical validation and advance GLO-1-targeted therapies toward clinical development. Other approaches should also utilize GLO-1 knockdown and overexpression models to examine subsequent biological effects on therapeutic outcome.

## 5 Conclusions

In this study, we investigated GLO-1 expression in a diverse range of cancer types and analyzed its role in cancer immunopathology. Furthermore, we analyzed the complex interplay between cancer immunotherapy and GLO-1 expression by examining its association with key immunotherapeutic biomarkers, including TMB, MSI, and a panel of ICPs. Our findings suggest that GLO-1 contributes to cancer progression by fostering an immunosuppressive microenvironment through MG-H1-mediated upregulation of the immune checkpoint programmed death ligand 1 (PD-L1). Future studies employing targeted modulation strategies and combination therapies could provide substantial clinical benefits.

## Data Availability

The original contributions presented in the study are included in the article/**Supplementary Material**. Further inquiries can be directed to the corresponding author.
