# Supplementary material for: Glyoxalase 1 gene expression in various types of cancer cells immunopathology: a pan-cancer analysis study
Source: Front Oncol. 2025 Jul 14;15:1610886. doi: 10.3389/fonc.2025.1610886 (PMC12301202; doi:10.3389/fonc.2025.1610886)
Supplement: Supplementary file 1 [file Table1.docx]

**Table S1:** Datasets utilized in this study, including sources, and tissue types.

| **Database** | **Data Type** | **Purpose** |
| --- | --- | --- |
| TCGA | The Cancer Genome Atlas | Contains genomic and clinical data from various human cancers. |
| TARGET | Therapeutically Applicable Research to Generate Effective Treatments | Focuses on pediatric cancers. |
| GTEx | Genotype-Tissue Expression project | Provides gene expression data from normal human tissues (used as healthy controls). |
| TIMER2.0 | Tumor Immune Estimation Resource 2.0/  Gene Expression Profiling Interactive Analysis 2.0 | Provides survival analysis, assessing the correlation between GLO-1 expression and both overall survival (OS) and disease-free survival (DFS)/ to investigate GLO-1 genetic alterations Samples were stratified into high and low GLO-1 expression groups based on the median expression value. |
| GEPIA2.0 |  |  |
| UALCAN | The University of ALabama at Birmingham CANcer data analysis Portal | Focused on breast, ovarian, and colon cancer datasets to examine the correlation between GLO-1 protein levels and clinical stages. |
| cBioPortal | Platform for study of cancer genomics data to incorporate complex molecular profiles with clinical outcomes. | To investigate GLO-1 genetic alterations, this resource was utilized to identify mutations within the GLO-1 gene. |
| SangerBox | The platform provides interactive customizable analysis tools (The correlation GLO-1 expression and a comprehensive panel of 47 ICP genes) | Copy number alteration data for GLO-1 to explore the interplay between GLO-1 expression and immune regulation within the tumor microenvironment, |
| GSCA/ CTRP | Drug Sensitivity Using the Gene Set Cancer Analysis/ Drug Sensitivity in Cancer and Cancer Therapeutics Response | Investigated the relationship between gene expression and drug sensitivity/ to assess the correlation between GLO-1 expression levels and susceptibility to small molecule drugs, as measured by IC50 values. |
